# The long non-coding RNA *ROCR* contributes to SOX9 expression and chondrogenic differentiation of human mesenchymal stem cells

**DOI:** 10.1242/dev.152504

**Published:** 2017-12-15

**Authors:** Matt J. Barter, Rodolfo Gomez, Sam Hyatt, Kat Cheung, Andrew J. Skelton, Yaobo Xu, Ian M. Clark, David A. Young

**Affiliations:** 1 Skeletal Research Group, Institute of Genetic Medicine, Newcastle University, Newcastle upon Tyne, NE1 3BZ, UK; 2 Musculoskeletal Pathology Group, Institute IDIS, Travesia choupana s/n, Hospital Clínico Universitario de Santiago, Santiago de Compostela, 15706, Spain; 3 Institute of Cancer and Genetics, School of Medicine, Cardiff University, Heath Park, Cardiff, CF14 4XN, UK; 4 Biomedical Research Centre, School of Biological Sciences, University of East Anglia, Norwich, NR4 7TJ, UK

**Keywords:** SOX9, LncRNA, Cartilage, Chondrogenesis, MSC, Epigenetics, Differentiation

## Abstract

Long non-coding RNAs (lncRNAs) are expressed in a highly tissue-specific manner and function in various aspects of cell biology, often as key regulators of gene expression. In this study, we established a role for lncRNAs in chondrocyte differentiation. Using RNA sequencing we identified a human articular chondrocyte repertoire of lncRNAs from normal hip cartilage donated by neck of femur fracture patients. Of particular interest are lncRNAs upstream of the master chondrocyte transcription factor SOX9 locus. SOX9 is an HMG-box transcription factor that plays an essential role in chondrocyte development by directing the expression of chondrocyte-specific genes. Two of these lncRNAs are upregulated during chondrogenic differentiation of mesenchymal stem cells (MSCs). Depletion of one of these lncRNAs, *LOC102723505*, which we termed *ROCR* (regulator of chondrogenesis RNA), by RNA interference disrupted MSC chondrogenesis, concomitant with reduced cartilage-specific gene expression and incomplete matrix component production, indicating an important role in chondrocyte biology. Specifically, SOX9 induction was significantly ablated in the absence of *ROCR*, and overexpression of SOX9 rescued the differentiation of MSCs into chondrocytes. Our work sheds further light on chondrocyte-specific SOX9 expression and highlights a novel method of chondrocyte gene regulation involving a lncRNA.

## Abstract (precis)

**Summary:** This study identified a chondrocyte repertoire of lncRNAs and discovered that *ROCR* (regulator of chondrogenesis RNA) is important for MSC chondrogenesis and cartilage gene expression by promoting the expression of SOX9.

## INTRODUCTION

Tens of thousands of long non-coding RNAs (lncRNAs) have been identified in the human genome through the use of RNA deep sequencing (RNA-Seq) (Iyer et al., 2015). lncRNAs are classified as >200-nucleotide RNAs that derive from both intergenic and overlapping protein-coding gene regions (Derrien et al., 2012). Detailed studies are beginning to ascribe functional roles for many of these lncRNAs, which appear to regulate numerous cell processes (Rinn and Chang, 2012). Indeed, lncRNAs have emerged as key regulators of gene expression transcriptionally and post-transcriptionally, acting through diverse mechanisms such as the regulation of epigenetic modifications and by acting as scaffolds for protein complex formation at gene loci (Rinn and Chang, 2012). lncRNAs display more tissue-specific expression patterns than protein-coding genes, and cell differentiation during development is particularly susceptible to experimental loss of lncRNAs (Derrien et al., 2012; Sauvageau et al., 2013; Fatica and Bozzoni, 2014). For example, lncRNAs play important roles in guiding limb development. In limb patterning, *HOTTIP* is required for specification of mesenchyme condensation sites through promotion of *HOXA* gene expression by a cis-regulatory mechanism, and *HOTAIR* is now also recognised for a similar trans-acting role in regulating *HOXD* gene expression during skeletal patterning (Wang et al., 2011; Li et al., 2013).

Little is known about the expression of lncRNAs in cartilage or in the development of the chondrocyte, the sole cartilage cell type. Chondrocytes develop from condensations of mesenchymal cells in a process known as chondrogenesis, which is essential for development of the endochondral skeleton (Onyekwelu et al., 2009). During chondrogenesis, cells of the mesenchyme commit to a chondrocyte differentiation programme then progress through multiple stages to specify the resting, proliferating and hypertrophic regions of the growth plate. They also constitute the articular cartilage at the ends of the long bones. This differentiation is a coordinated process determined by temporal and spatial expression of multiple growth factors and dependent on the specific activity of the HMG-box transcription factor SOX9 (Akiyama, 2008). SOX9 controls the expression of numerous chondrocyte genes, including its co-factors L-SOX5a and SOX6, and extracellular matrix genes such as type II collagen and the proteoglycan aggrecan. Experimental loss of SOX9 abrogates limb development in mice (Akiyama, 2008; Akiyama and Lefebvre, 2011) and mutations in the *SOX9* coding sequence lead to the skeletal malformation syndrome campomelic dysplasia (CD) (Foster et al., 1994; Wagner et al., 1994). DNA alterations around the *SOX9* locus can also lead to CD, highlighting the complex regulatory mechanisms governing *SOX9* expression (Foster et al., 1994; Wagner et al., 1994).

*SOX9* is found in a gene desert on chromosome 17, as is common for developmental transcription factors, surrounded by many potential regulatory regions. However, the cellular mechanisms for regulating *SOX9* are not fully established. Analyses of CD patient chromosomal rearrangements and promoter yeast artificial chromosome transgenes suggest that certain chondrogenesis-specific enhancers lie in a region between 50 kb and 350 kb upstream of *SOX9* (Foster et al., 1994; Wagner et al., 1994; Wunderle et al., 1998; Gordon et al., 2009). SOX9 also specifies the fate of other lineages, including Sertoli cells, neural stem cells, pancreas progenitor cells and neural crest, neuronal, glial, heart valve, gut and kidney cells (Pritchett et al., 2011). Again, tissue-specific enhancers have been demonstrated to regulate the expression in some of these tissues (Gordon et al., 2009).

cDNA cloning methods and *in silico* genome analysis have established that numerous expressed sequence tags and predicted transcripts are localised to these enhancer regions upstream of *SOX9* but it is unclear which are expressed in particular tissues and whether any have a functional role in chondrocytes. We established a chondrocyte repertoire of lncRNAs and confirmed the presence of a number of transcripts around the *SOX9* locus by whole transcriptome analysis of human articular cartilage RNA by RNA-Seq. We discovered a novel cartilage-specific 4-exon lncRNA corresponding to a 3-exon RefSeq transcript *LOC102723505* (*LINC02095*, Ensembl transcript *ENST00000430908*) 94 kb upstream of *SOX9*, which we termed *ROCR* (regulator of chondrogenesis RNA). This lncRNA is required for successful differentiation of mesenchymal stem cells (MSCs) into chondrocytes where it appears to contribute to *SOX9* expression. Thus, we have identified a previously unknown mechanism of *SOX9* regulation involving a chondrocyte-specific lncRNA.

## RESULTS

### Human articular chondrocyte lncRNAs

**Fig. 1.**
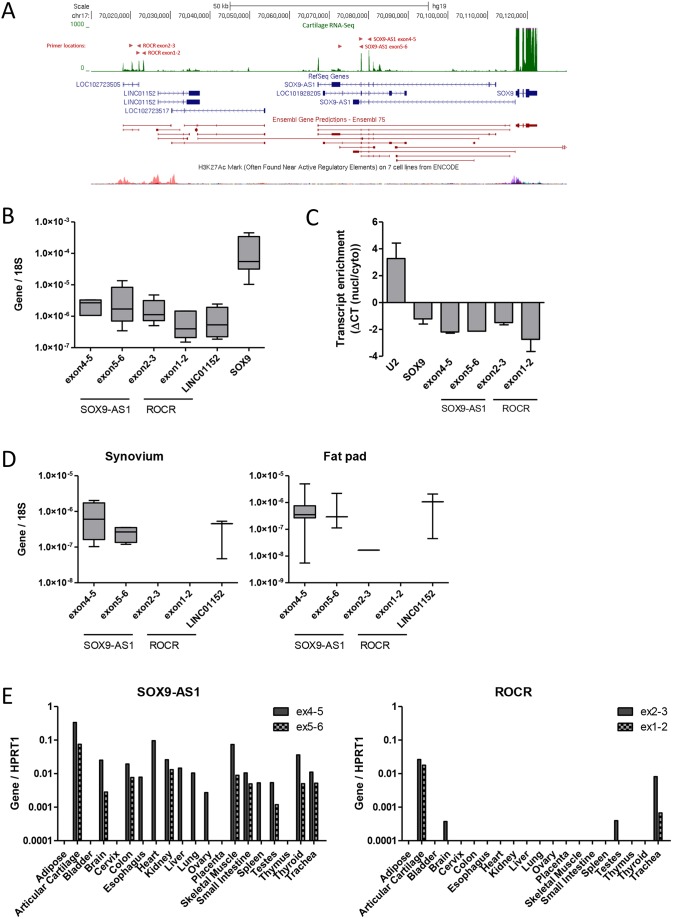
**Expression of lncRNAs from the *SOX9* locus.** (A) UCSC genome browser schematic of cartilage RNA-Seq reads aligned to the human genome with RefSeq gene annotations, Ensembl gene predictions and active H3K27Ac chromatin marks. Reads are pooled from six neck of femur (NOF) fracture cartilage donors. Primer locations are indicated by red arrowheads. (B) Expression of *SOX9* locus lncRNAs and *SOX9* in RNA extracted from OA cartilage measured by real-time RT-PCR normalised to 18S. Values are mean±s.e.m. of data pooled from five separate donors. (C) Subcellular localisation of *SOX9-AS1* and *ROCR* in comparison with small nuclear RNA *U2* and *SOX9* mRNA pooled from two OA HAC donors. Values are mean±s.e.m. of ΔCT between an equal fraction of nuclear and cytoplasmic RNA. (D) Expression of *SOX9-AS1* and *ROCR* in RNA extracted from OA synovium and joint fat pad. Values are mean±s.e.m. of data pooled from eight separate synovium and fat pad donors. (E) Expression of *SOX9-AS1* and *ROCR* in an RNA tissue panel. Values are the technical mean of data pooled from three donors per tissue.

RNA-Seq was performed on normal human hip articular chondrocyte RNA obtained from female neck of femur (NOF) fracture patients to establish the adult chondrocyte transcriptome and its complement of lncRNAs (6 samples; median age=76 years). Of the 46,087 transcripts identified [fragments per kilobase of exon per million fragments mapped (FPKM)>1], 813 were annotated as lncRNAs (Table S2). Examination of cartilage RNA-Seq reads uploaded to the UCSC genome browser identified processed transcripts upstream of the *SOX9* locus on chromosome 17, with robust expression of transcripts corresponding to *SOX9-AS1* and *LOC102723505* (Fig. 1A), exon/intron boundaries, and evidence of transcript start and end sites using cap analysis gene expression (CAGE) and PolyA-Seq data (ENCODE Project Consortium, 2012; Flicek et al., 2014) (Fig. S1). Proximal to the *SOX9* locus transcript, variants of *SOX9-AS1* were detected partially corresponding to Refseq and predicted Ensembl transcripts. 94 kb upstream of *SOX9* we detected a novel 4-exon variant of an existing 3-exon RefSeq transcript *LOC102723505.* We designated the 3-exon *LOC102723505* as *ROCR* (regulator of chondrogenesis RNA) transcript variant 1 and the novel 4-exon isoform *ROCR* transcript variant 2. We noted the presence of chromatin features of actively transcribed genes, such as histone H3 lysine 4 trimethylation (H3K4me3), at the presumed *ROCR* promoter and enhancer-like signatures based on histone lysine 27 acetylation (H3K27ac) states from ENCODE chromatin state data (Fig. 1A, Fig. S1) (Ernst et al., 2011). The *ROCR* locus is also notable for the expression of an additional lncRNA, *LINC01152*, albeit at very low levels in cartilage. In comparison with other coding transcripts, *SOX9-AS1* and *ROCR* were moderately expressed in cartilage with FPKM in the range of 5-15, approximately 10% of the level of *SOX9* itself (Table S2).

We confirmed the expression of *SOX9-AS1* and *ROCR* in human articular cartilage by qRT-PCR with two assays per transcript targeted to different exons (Fig. 1B). The *ROCR* exon1-2 assay detects only transcript variant 2. Rapid amplification of cDNA ends (RACE) confirmed the presence of this novel 4-exon 574-base *ROCR* transcript (variant 2) in cartilage (Fig. S2). We were also able to identify this *ROCR* variant by subsequent analysis of RNA-Seq data from knee cartilage RNA (Dunn et al., 2016). The majority of lncRNAs are considered to have nuclear functions and are often found enriched in the nucleus (Quinodoz and Guttman, 2014). In contrast to the nuclear enrichment of the small nuclear RNA U2, we found that both *SOX9-AS1* and *ROCR* were enriched in the cytoplasm, comparable with the localisation of the *SOX9* transcript itself (Fig. 1C). RNA fluorescence *in situ* hybridisation (RNA-FISH) analysis of *ROCR* in human articular chondrocytes (HACs) was unsuccessful owing to the relatively low expression and short transcript sequence, which limited the design of sufficient singly labelled Stellaris RNA-FISH probes (data not shown). *In silico* analysis indicates a lack of coding potential for both *SOX9-AS1* and *ROCR*, with the existence of only very short open reading frames (ORF Finder) and codon substitution rates indicative of noncoding transcripts (CPAT, CPC and PhyloCSF) (Fig. S3). *SOX9* is expressed in a variety of tissues but lncRNAs are reported to be more tissue specific (Derrien et al., 2012). Accordingly, we examined expression of *SOX9-AS1* and *ROCR* in additional joint tissues extracted from osteoarthritis (OA) patients. *SOX9-AS1* was also expressed in synovium and fat pad tissue but *ROCR* was largely undetected indicating that it might be specific to cartilage in the joint (Fig. 1D).

We further examined transcript expression bioinformatically using publicly available cell and tissue RNA-Seq databases. Reads corresponding to *SOX9-AS1* were found in numerous cells types in both Human Protein Atlas (http://www.proteinatlas.org/) and Illumina BodyMap (ArrayExpress accession: E-MTAB-513; http://www.ebi.ac.uk/arrayexpress) sequence data (Table S3) (ENCODE Project Consortium, 2012; Krupp et al., 2012; Fagerberg et al., 2014; Flicek et al., 2014). Reads corresponding to the three exons of *ROCR* transcript variant 1 were found in pancreas and salivary gland tissue samples in the Human Protein Atlas RNA-Seq data and in breast tissue samples sequenced in the Illumina BodyMap data. Consistent with this analysis, further examination of expression by qRT-PCR across a 20-tissue RNA panel again identified the presence of *SOX9-AS1* transcripts in a number of tissues (Fig. 1E), albeit in fewer tissues than *SOX9* itself (Fig. 4). In contrast, detection of the novel *ROCR* transcript variant 2 was limited to chondrocytes alone (Fig. 1F), and the *ROCR* transcript variant 1 was additionally detected in brain and testis.

### *SOX9* locus lncRNA expression in MSC differentiation

**Fig. 2.**
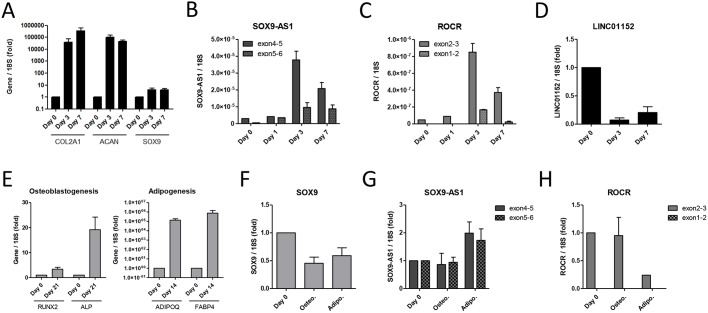
**Expression of lncRNAs during MSC differentiation.** (A) Expression of the indicated genes in RNA extracted from MSCs undergoing chondrogenic differentiation at the indicated time points between Day 0 and Day 7. (B-D) Expression of *SOX9-AS1* (B), *ROCR* (C) and *LINC01152* (D) during MSC chondrogenic differentiation. (E) Expression of the indicated genes in RNA extracted from MSCs undergoing osteoblastogenic and adipogenic differentiation. (F-H) Expression of *SOX9* (F), *SOX9-AS1* (G) and *ROCR* (H) during MSC osteoblastogenic (Osteo.) and adipogenic (Adipo.) differentiation. Values are mean± s.e.m. of data pooled from three or four MSC donors.

Considering the proximity of these transcripts to *SOX9* and the potential chondrocyte specificity of *ROCR*, we sought to establish whether *SOX9-AS1* and *ROCR* were regulated during chondrocyte development. Accordingly, we characterised expression of these lncRNAs using a robust transwell MSC chondrogenesis method, which produces a uniform cartilage disc with rapid and substantial induction of chondrocyte gene expression, albeit including the expression of chondrocyte hypertrophy genes, thus differing from articular cartilage (Fig. 2A) (Murdoch et al., 2007). *SOX9* expression is upregulated during chondrogenesis. Similarly, the expression of both *SOX9-AS1* and *ROCR* was induced during MSC chondrogenesis, paralleling the kinetics of *SOX9* expression (Fig. 2B,C). In contrast, *LINC01152*, a potential testis-specific lncRNA (D43770 Genbank ID), was downregulated during MSC chondrogenesis (Fig. 2D) (Ninomiya et al., 1996). Interestingly, RACE for MSC RNA identified a further 624-base isoform with an alternative first exon, which we termed *ROCR* transcript variant 3 (Fig. S1), situated in a bidirectional promoter locus with *LINC01152*.

MSCs are capable of tri-lineage differentiation into chondrocytes, osteoblasts and adipocytes, dependent on specific differentiation factors (Pittenger et al., 1999). We differentiated MSCs into osteoblasts and adipocytes by established methods and confirmed the expression of the osteoblast-specific markers alkaline phosphatase (*ALPL*) and *RUNX2*, and the adipocyte-specific genes adiponectin and *FABP4* (Fig. 2E). *SOX9* was not upregulated during osteoblastogenesis or adipogenesis (Fig. 2F). Similarly, *SOX9-AS1* and *ROCR* were not upregulated during MSC osteoblastogenesis (Fig. 2G,H). *SOX9-AS1* was induced during MSC adipogenesis, in contrast to *ROCR*, but not to the level of chondrogenesis (Fig. 2G).

### Role of *ROCR* in MSC chondrogenesis

**Fig. 3.**
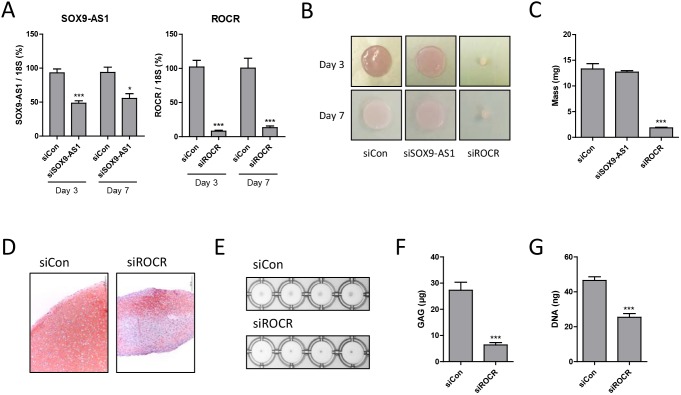
**Effect of lncRNA depletion on MSC chondrogenic differentiation.** (A-D) MSCs were transfected for 2 days with *SOX9-AS1* or *ROCR*-targeting or non-targeting control (siCon) siRNA prior to chondrogenic differentiation in hanging transwell inserts. (A) *SOX9-AS1* and *ROCR* expression in RNA extracted from Day 3 and Day 7 chondrogenic discs. Expression is presented as a percentage of non-targeting control levels. (B) Representative Day 3 and 7 chondrogenic discs. (C) Wet mass of Day 7 chondrogenic discs. (D) Representative Safranin O staining of Day 7 chondrogenic discs. (E-G) MSCs were transfected for 1 day with *ROCR*-targeting or non-targeting control siRNA prior to chondrogenic differentiation in a V-bottom 96-well plate. (E) Representative Day 7 chondrogenic pellets. (F) GAG levels assayed by DMB assay in Day 7 chondrogenic pellets. (G) DNA quantification by PicoGreen assay in Day 7 chondrogenic pellets. Values are mean±s.e.m. of data pooled from three (A-D) or four (E-G) MSC donors. *P<0.05; ***P<0.001 for lncRNA siRNA versus non-targeting siRNA. Significant differences between sample groups were assessed by one-way analysis of variance followed by the Bonferroni post-hoc test for multiple comparisons or a two-tailed Student's *t*-test was performed for single comparisons.

*SOX9-AS1* and *ROCR* were both upregulated during chondrogenesis, with a profile similar to *SOX9*; therefore, we addressed their potential role during MSC chondrogenic differentiation by specific RNA interference (RNAi)-mediated depletion (Fig. 3A). Reduction of *SOX9-AS1* expression had no effect on development of a cartilaginous disc (Fig. 3B,C). However, depletion of *ROCR* prevented disc formation (Fig. 3B) and caused a significant reduction in wet mass (Fig. 3C). Consistent with the disruption of disc formation following *ROCR* RNAi, matrix deposition in the form of glycosaminoglycan (GAG) polyanions was also reduced (Fig. 3D). In case the transwell chondrogenesis method was particularly susceptible to experimental manipulation, we also performed the traditional pellet chondrogenesis method and again found that *ROCR* was required for pellet development (Fig. 3E). Analysis of extracted sulphated GAG levels again indicated that *ROCR* is required for matrix GAG production (Fig. 3F). In addition, *ROCR* depletion reduced DNA levels suggesting it was required for MSC proliferation during the early stages of chondrocyte differentiation (Fig. 3G) (Murdoch et al., 2007).

**Fig. 4.**
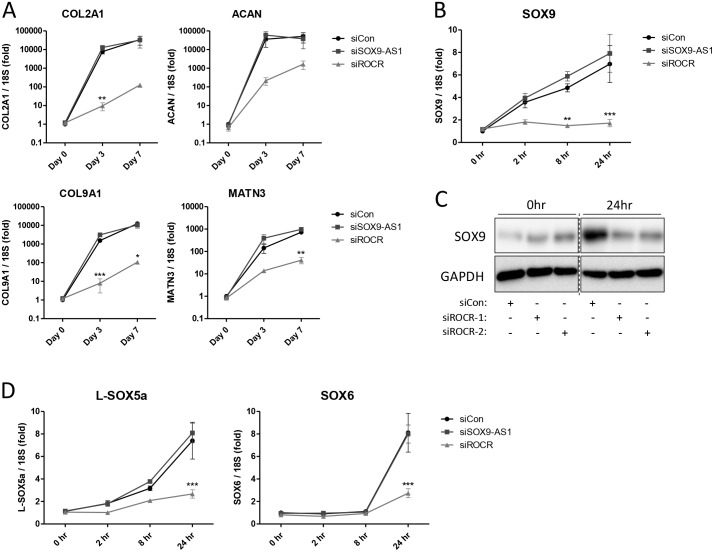
**Effect of lncRNA depletion on MSC chondrogenic gene expression.** (A) MSCs were transfected for 2 days with *SOX9-AS1* or *ROCR*-targeting or non-targeting control (siCon) siRNA prior to chondrogenic differentiation in hanging transwell inserts. RNA was extracted and expression of the indicated genes measured by real-time RT-PCR. (B-D) MSCs were transfected for 1 day with *SOX9-AS1* or *ROCR*-targeting or non-targeting control siRNA prior to chondrogenic differentiation in a V-bottom 96-well plate for up to 24 h. RNA and protein was extracted and expression of *SOX9* mRNA (B) or protein (C) measured by real-time RT-PCR or immunoblotting, respectively. (D) Expression of *L-SOX5a* and *SOX6*. Values are mean±s.e.m. of data pooled from three (A) or four (B-D) MSC donors. *P<0.05; **P<0.01; ***P<0.001 for lncRNA siRNA versus non-targeting siRNA. Significant differences between sample groups were assessed by one-way analysis of variance followed by the Bonferroni post-hoc test for multiple comparisons or a two-tailed Student's *t*-test was performed for single comparisons.

Examination of chondrocyte gene expression following *SOX9-AS1* and *ROCR* RNAi indicated that depletion of *ROCR* also significantly abrogated the induction of cartilage extracellular matrix (ECM) genes including *COL2A1* and *ACAN* (Fig. 4A). SOX9 is essential for cartilage matrix gene expression, so we assessed the impact of depletion of *SOX9-AS1* and *ROCR* at earlier time points in the chondrogenesis time course. Following *ROCR* depletion, *SOX9* mRNA (Fig. 4B) and protein (Fig. 4C) was significantly reduced after 1 day of MSC differentiation, and at even earlier time points the upregulation of *SOX9* expression during MSC chondrogenesis was lost following *ROCR* depletion suggesting a crucial role for *ROCR* in *SOX9* induction. During chondrogenesis SOX9 is required for expression of *SOX5* and *SOX6*, which subsequently cooperate with SOX9 in directing chondrocyte gene expression (Akiyama et al., 2002). *ROCR* depletion also prevented the upregulation of the SOX9 target genes *SOX5* and *SOX6*, which occurred after *SOX9* induction (Fig. 4D).

To complement the role identified by RNAi for *ROCR* in MSC chondrogenesis and *SOX9* expression, we also used an LNA GapmeR approach to deplete cellular *ROCR* levels (Fig. S5). Again, the loss of *ROCR* resulted in a significant reduction in matrix GAG formation during MSC mini-pellet chondrogenesis with concomitant reduction in *SOX9* and matrix gene expression (Fig. S5). *ROCR* transcript variants 2 (HAC) and 3 (MSCs) were cloned and overexpressed in MSCs and HAC by lentiviral transduction (Fig. S6). Overexpression of *ROCR* had no effect on SOX9 expression or induction of the cartilage ECM genes *COL2A1* and *ACAN* during MSC chondrogenesis (Fig. S6A). Overexpression of *ROCR* had no effect on SOX9 expression in HAC (Fig. S6B).

### Specificity of *ROCR* function to chondrogenesis

**Fig. 5.**
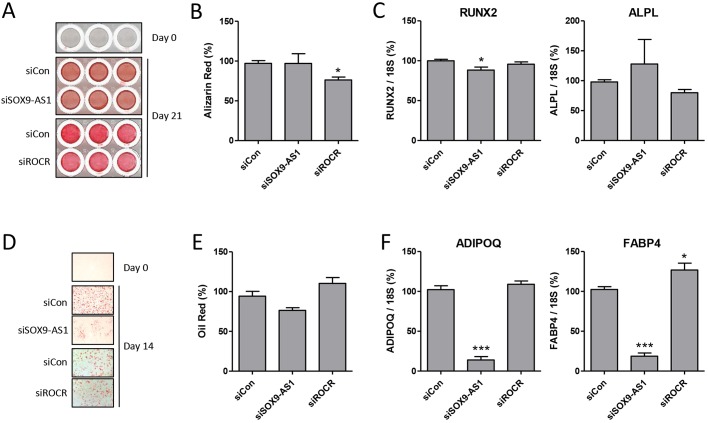
**Effect of lncRNA depletion on MSC osteoblastogenic and adipogenic differentiation.** (A-C) MSCs were transfected for 2 days with *SOX9-AS1* or *ROCR*-targeting or non-targeting control (siCon) siRNA prior to osteoblastogenic differentiation. (A) Representative matrix mineralisation assayed by Alizarin Red staining after 21 days. (B) Quantification of Alizarin Red staining. (C) RNA was extracted and expression of the indicated genes at Day 7 measured by real-time RT-PCR. (D-F) MSCs were transfected for 2 days with *SOX9-AS1* or *ROCR*-targeting or non-targeting control siRNA prior to adipogenic differentiation. (D) Representative fat droplet generation assayed by Oil Red O staining after 14 days. (E) Quantification of Oil Red O staining. (F) RNA was extracted and expression of the indicated genes at Day 7 measured by real-time RT-PCR. Values are mean±s.e.m. of data pooled from four MSC donors. *P<0.05; ***P<0.001 for lncRNA siRNA versus non-targeting siRNA. Significant differences between sample groups were assessed by one-way analysis of variance followed by the Bonferroni post-hoc test for multiple comparisons or a two-tailed Student's *t*-test was performed for single comparisons.

The above data suggested that *ROCR* is important for MSC chondrogenesis. We sought to establish whether the role of *ROCR* was specific to chondrocyte development consistent with its restricted expression profile. Accordingly, we also performed *SOX9-AS1* and *ROCR* RNAi during MSC osteoblastogenesis and adipogenesis. Depletion of *ROCR* during osteoblast differentiation caused a partial decrease in matrix mineralisation (Fig. 5A,B), but no significant impact on *RUNX2* or *ALPL* expression (Fig. 5C). During MSC adipogenesis *ROCR* depletion had little effect, whereas *SOX9-AS1* depletion partially reduced fat droplet generation (Fig. 5D,E) and significantly decreased MSC adipogenic gene expression (Fig. 5F).

**Fig. 6.**
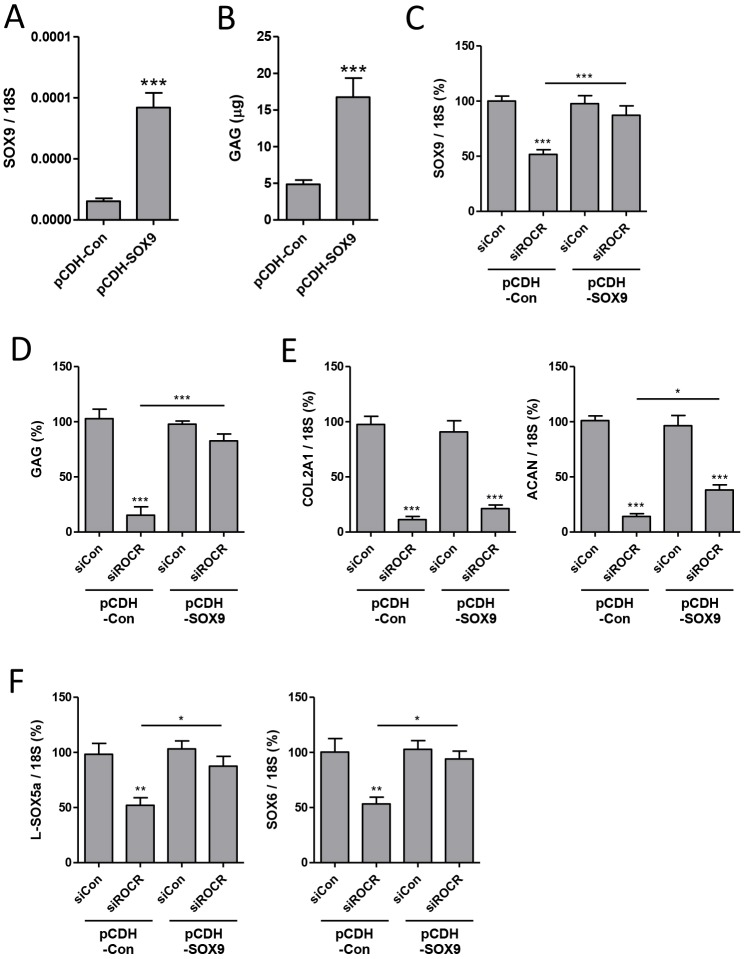
**Effect of SOX9 overexpression in combination with *ROCR* depletion on MSC chondrogenic differentiation and gene expression.** MSCs were transduced with SOX9 or control lentivirus (pCDH) for 1 day then transfected for 1 day with *ROCR*-targeting or non-targeting control (siCon) siRNA prior to chondrogenic differentiation in a V-bottom 96-well plate. (A) Expression of *SOX9* in only non-targeting control siRNA pellets at Day 3. (B) GAG levels assayed by DMB assay in only non-targeting control siRNA pellets at Day 7. (C) Expression of *SOX9* at Day 3. (D) GAG levels assayed by DMB assay at Day 7. (E,F) Expression of the indicated genes at Day 3. (C-F) Expression is presented as a percentage of non-targeting control levels for cells transduced with each virus. Values are mean±s.e.m. of data pooled from four MSC donors. *P<0.05; **P<0.01; ***P<0.001 for lncRNA siRNA versus non-targeting siRNA, or for SOX9 versus control lentivirus where indicated above the chart. Significant differences between sample groups were assessed by one-way analysis of variance followed by the Bonferroni post-hoc test for multiple comparisons or a two-tailed Student's *t*-test was performed for single comparisons.

SOX9 is essential for chondrogenesis and as lncRNAs can contribute to the expression of neighbouring genes (Vance and Ponting, 2014) we reasoned that the primary role of *ROCR* is to promote *SOX9* expression. Accordingly, overexpression of SOX9 would be expected to rescue the chondrogenesis impairment caused by *ROCR* depletion. Lentiviral overexpression of SOX9 successfully enhanced MSC chondrogenesis (Fig. 6A,B). By overexpressing SOX9 and thereby returning the levels of SOX9 to those of control (Fig. 6C) the significant reduction of cartilage matrix GAG levels following depletion of *ROCR* was almost fully reversed (Fig. 6D). Reduction of *COL2A1* and *ACAN* by *ROCR* depletion was partially reversed by overexpression of SOX9 (Fig. 6E,F), and the levels of *L-SOX5a* and *SOX6* were completely rescued (Fig. 6G,H).

## DISCUSSION

In this study, we established a panel of lncRNAs in normal human articular cartilage and identified two transcripts upstream of the *SOX9* locus that were upregulated during MSC chondrogenesis. One of these, *ROCR*, is a functional cartilage-restricted lncRNA that appears to be important for chondrocyte differentiation where it might facilitate the induction of *SOX9* itself. This study established that a lncRNA contributes to SOX9 expression during differentiation of MSCs into chondrocytes, thereby furthering our understanding of the key regulatory elements contained upstream of the *SOX9* promoter.

SOX9 is the master transcription factor governing chondrocyte development, as confirmed by genetic studies (Akiyama et al., 2002). Regulation of SOX9 occurs at both the transcriptional and post-transcriptional levels. Phosphorylation of SOX9 regulates its DNA-binding activity and subcellular localisation, and numerous other interactions regulate SOX9 stability and facilitate its transcriptional activity (Kawakami et al., 2006; Akiyama, 2008). At the transcriptional level, induction of *SOX9* occurs rapidly during mesenchyme condensation in cartilage development both *in vivo* and *in vitro* (Wright et al., 1995; Sekiya et al., 2002), a process regulated by an interplay between growth factor signals and cell-cell interactions (Chimal-Monroy et al., 2003; Yoon et al., 2005). Our data indicated that during *in vitro* chondrogenesis a lncRNA, *ROCR*, is also important for this process.

### lncRNAs in chondrocytes and chondrogenesis

A number of lncRNAs have key roles in stem cell differentiation, including *RMST* in neuronal differentiation, *Braveheart* in cardiac differentiation and *lnc-RAP1-10* in adipocyte differentiation (Klattenhoff et al., 2013; Ng et al., 2013; Perry and Ulitsky, 2016). Previously identified lncRNAs with a potential role in cartilage development include *DA125942* and *LncRNA-HIT* (Maass et al., 2012; Carlson et al., 2015). *DA125942*, a lncRNA transcribed from the *CISTR-ACT* locus interacts *in cis* with *PTHLH* and *in trans* with *SOX9* to organise chromatin structure and promote transcription in cartilage (Maass et al., 2012). No direct role for the lncRNA in chondrogenesis was explored although the lncRNA locus was active during mouse limb bud development. *LncRNA-HIT*, expressed in mouse limb mesenchyme from the *Hoxa* gene locus, is able to bind and regulate DNA regions surrounding a number of cartilage genes including the *Hoxa* genes themselves (Carlson et al., 2015). *LncRNA-HIT* may activate gene expression by binding to the p100/CBP complex and it contributes to micromass chondrogenic differentiation of murine MSCs. Interestingly, we detected no RNA expression from the *CISTR-ACT* locus in our human cartilage RNA-Seq data and the conserved regions of *LncRNA-HIT* in human corresponded to an isoform of *HOXA13* with an extended 3′UTR rather than a lncRNA. It is possible that these lncRNAs might be developmental stage or MSC specific. The lncRNA *DANCR* may also promote chondrogenic differentiation of synovium-derived MSCs in concert with SOX4 (Zhang et al., 2015). Two recent reviews elaborate on the roles of these lncRNAs during chondrogenesis (Huynh et al., 2017; Lefebvre and Dvir-Ginzberg, 2017).

*SOX9* is located in a ∼2 Mb gene desert on chromosome 17 in humans and lncRNA *ROCR* is expressed from a locus 94 kb upstream of *SOX9*. Chromosomal rearrangements within this region are associated with CD, a skeletal malformation syndrome, and Pierre Robin sequence (PRS), a craniofacial disorder. Such disruptions can occur in regions up to and greater than 1 Mb upstream of *SOX9* (Gordon et al., 2009). Characterisation of these DNA alterations has indicated the presence of enhancer regions linked to the regulation of *SOX9* expression. Breakpoints causing more severe forms of CD are found more proximal to *SOX9* at locations 50-375 kb upstream (Leipoldt et al., 2007). Transgene and reporter experiments have also indicated that sequences in these locations are able to drive gene expression *in vivo* (Gordon et al., 2009). More recent analysis confirmed the presence of a murine enhancer element at −70 kb (−62 kb in human) capable of regulating *SOX9* expression in a number of tissues (Mead et al., 2013), and three further enhancers with prominent activity in chondrocytes at −84 kb, −195 kb and −250 kb in mice (Yao et al., 2015).

The *ROCR* locus sits within these enhancer regions and it is attractive to suggest that the lncRNA might contribute to the regulation of *SOX9 in vivo*. Indeed, functional lncRNAs have been found to be enriched in genomic regions surrounding key developmental transcription factors (Ørom et al., 2010; Ulitsky et al., 2011). In addition to skeletal malformations, patients with CD often show XY sex reversal, with additional clinical features such as hearing loss, developmental delay, and occasional heart defects (Mansour et al., 2002). Consistent with this, genetic ablation of *Sox9* in mice disrupts the differentiation of cells in the heart, central nervous system, testis, pancreas, gut and inner ear (Gordon et al., 2009). Tissue-specific enhancers regulate the expression of *SOX9*, for example the testis enhancer TES at −10 kb, and our analysis suggested that *ROCR* is restricted to certain cell types – cartilage, brain and testis – whereas *ROCR* variant 2 was only detected in cartilage. However, our work focussed on RNA extracted from aged NOF and OA tissue and further work is required to confirm the expression of *ROCR* in normal healthy tissues. In combination with tissue-specific enhancers *ROCR* might be required for the tightly coordinated spatiotemporal expression of *SOX9* during development. The expression level of *SOX9* in cartilage was one or two orders of magnitude higher than other tissues (Fig. S4) and we reasoned that *ROCR* might also contribute to the magnitude of *SOX9* expression. But, in contrast to its role in chondrogenesis, we found no significant contribution by *ROCR* to *SOX9* expression levels in adult articular chondrocytes (Fig. S7A). The role of *ROCR* in *SOX9* expression might be in response to cues during chondrogenesis that are not present in cultured HAC, and ROCR might additionally regulate other genes/proteins. The induction of both *SOX9-AS1* and *ROCR* paralleled the expression of *SOX9*. The activity of the aforementioned −70 kb, −84 kb and −195 kb *SOX9* upstream enhancers is dependent on SOX9 in differentiated chondrocytes (Yao et al., 2015). Prior to the onset of chondrogenesis, SOX9 overexpression in MSCs did not significantly induce *ROCR* expression (Fig. S7B), but we cannot rule out the possibility that SOX9 promotes the expression of *ROCR* during chondrogenesis, or in adult chondrocytes. Despite knockdown of *ROCR* reducing SOX9 expression and cartilage gene expression in MSCs, reciprocal overexpression of *ROCR* had no effect. Overexpression from an artificial plasmid transcription start site is not entirely analogous to endogenous *ROCR* expression with potential alteration to secondary structure formation and cellular localisation of the RNA.

During skeletogenesis MSC condensation initiates the formation of multipotent osteochondroprogenitors the lineage fate of which is then determined by the combination of growth factor signals received. *ROCR* is only upregulated during chondrogenesis, not osteoblastogenesis, suggesting a key role in directing MSCs toward the chondrocyte lineage. Consistent with this, only a minor impact of *ROCR* depletion was observed during MSC osteoblastogenesis in contrast to its key requirement during chondrogenesis. During osteochondroprogenitor differentiation SOX9 has antagonistic effects on the osteoblast transcription factor RUNX2 in determining the specific differentiation into their respective chondrocyte and osteoblast lineages (Zhou et al., 2006). Owing to the lack of induction of *ROCR* during osteoblastogenesis, no effect would be expected. Interestingly, depletion of *SOX9-AS1* significantly reduced the expression of adipogenic marker genes, confirming the efficacy of the *SOX9-AS1* depletion and, given the role of SOX9 in adipogenic differentiation, suggests that *SOX9-AS1* also contributes to the differentiation (Stockl et al., 2013).

### Putative lncRNA function

We demonstrated that returning SOX9 levels to normal by overexpression could reverse the impaired chondrogenesis phenotype caused by depletion of *ROCR*. This indicated that SOX9 can largely replace *ROCR* during MSC chondrogenesis as *SOX9* expression was sufficient to produce the cartilage matrix. Thus, suggesting *ROCR* is indirectly needed in chondrogenesis to establish the correct level of *SOX9* expression in MSCs during differentiation. Both silencing and activating roles have been demonstrated for lncRNAs. *XIST* establishes X chromosome inactivation, whereas *RMST* facilitates SOX2 binding to promoter regions of neurogenic transcription factors (Vance and Ponting, 2014). In some cases, enhancer regions and the process of transcription at the lncRNA locus facilitate downstream gene expression rather than the lncRNA transcript itself (Engreitz et al., 2016). Our knockdown experiments indicate that *ROCR* transcript is functional, and the *ROCR* locus is considerably upstream from SOX9 (94 kb), but we cannot rule out the possibility that the *ROCR* locus might also function as an enhancer. Many of the identified functional lncRNA actions occur in the nucleus; however, *ROCR* appears to reside more in the cytoplasm than nucleus, indicating an indirect regulation of SOX9. Our coding analysis indicated that *ROCR* is unlikely to code for any significant peptide transcript, suggesting a role for the RNA in the cytoplasm. A number of cytoplasmic lncRNAs can regulate mRNA half-life and translation. *TINCR* is induced during epidermal differentiation and is required for stability of differentiation mediators (Kretz et al., 2013) and antisense *Uchl1* lncRNA promotes translation of *Uchl1* in mouse (Carrieri et al., 2012). Other factors also contribute to cartilage gene expression, such as SP1 and forkhead/winged-helix domain (FOX) proteins, and this could account for why, despite normal GAG levels, the expression of *COL2A1* and *ACAN* was not completely restored during rescue by SOX9, again suggesting an indirect effect of *ROCR* (Liu et al., 2016). Or this might simply reflect the difference in sampling time for gene expression in relation to matrix GAG measurement. Almost all lncRNAs function through association with protein partners and, accordingly, RNA pulldown methods are commonly used to identify such interactions (Yang et al., 2015).

Conservation of lncRNAs across species is low, with less than 10% of all lncRNAs exhibiting regions of conservation compared with random control regions (Iyer et al., 2015), but there are key examples of conserved lncRNAs with crucial roles in mouse development having human counterparts (Sauvageau et al., 2013). By conducting a homology search for a mouse orthologue of *ROCR*, we identified a predicted noncoding RNA transcript (NR_024085/BC006965) with sequence similarity to exon 2 of *ROCR* transcript variant 1 (exon 3 of variants 2 and 3), but little mammalian sequence conservation in general (Fig. S8). Importantly, the transcripts are in syntenic regions (containing *SOX9*) of human chromosome 17q24 and mouse 11qE2. By real-time RT-PCR of mouse cartilage RNA we have now confirmed the expression of a murine multiple exon version of *ROCR* (Fig. S8). Further work will establish whether the murine transcript is regulated during chondrogenesis and contributes to chondrocyte development.

## Conclusions

The cartilage transcriptome contains many lncRNA transcripts many of which may have important functions in cartilage biology. Our identification of cartilage lncRNAs complements the previous identification of inflammation-induced lncRNAs in chondrocytes (Pearson et al., 2016). This panel of chondocyte lncRNAs is specific to human aged hip cartilage and further work should establish the expression of lncRNAs specific to different zones of articular cartilage, as well as growth plate cartilage and to establish the impact of weight bearing, age and disease such as OA. Functional analysis indicated that *ROCR* was induced during chondrogenic differentiation and played an important role in the induction of *SOX9* and, as a result, cartilage gene expression. Because SOX9-expressing cells are progenitors for numerous tissues, identifying chondrocyte-specific regulatory elements might aid our understanding of differentiation of chondrocytes from MSCs, which could be potentially useful in chondrocyte tissue-engineering applications.

## MATERIALS AND METHODS

### Human tissue isolation

Normal human articular cartilage was obtained from patients undergoing joint replacement surgery due to intracapsular neck of femur (NOF) fracture. OA human articular cartilage was obtained from knee joint replacement operations on patients diagnosed with osteoarthritis (OA). Synovium and infrapatellar fat pad were also collected from the knee of OA patients. All tissue was obtained with informed consent and ethics committee approval from the Newcastle and North Tyneside Health Authority. Scoring, extraction and patient information for the NOF samples are detailed in Xu et al. (2012). Briefly, joints were inspected macroscopically and scored by a blinded experienced orthopaedic surgeon to identify normal NOF cartilage. Cartilage, all zones, was collected within 2 h of surgery and stored at −80°C prior to RNA extraction.

### Human bone marrow MSC culture

Human bone marrow MSCs (from seven donors, 18-25 years of age) were isolated from human bone marrow mononuclear cells (Lonza Biosciences) and cultured and phenotype-tested as described previously (Barter et al., 2015). Experiments were performed using cells between passage 2 and 7, and all experiments were repeated with cells from three or four donors.

### Chondrogenic differentiation

MSCs were re-suspended in chondrogenic culture medium consisting of high-glucose DMEM containing 100 µg/ml sodium pyruvate, 10 ng/ml TGFβ3, 100 nM dexamethasone, 1× ITS-1 premix, 40 µg/ml proline and 25 µg/ml ascorbate-2-phosphate. MSCs (5×105 in 100 µl medium) were pipetted onto 6.5-mm diameter, 0.4-µm pore size polycarbonate Transwell filters (Merck Millipore), centrifuged at 200 ***g*** for 5 min, then 0.5 ml of chondrogenic medium was added to the lower well as described previously (Murdoch et al., 2007; Barter et al., 2015). For V-bottom 96-well-plate pellet chondrogenesis, 5×10^4^ MSCs in 150 µl chondrogenic medium were pipetted into a UV-sterilised V-bottom 96-well plate and centrifuged at 500 ***g*** for 5 min. Media were replaced every 2 or 3 days for up to 7 days.

### Osteoblast and adipocyte differentiation

MSCs were plated in 96-well plates at a density of 15,000/cm^2^ for 24 h then media were replaced with either osteoblastogenic culture medium consisting of DMEM supplemented with 10% (v/v) foetal bovine serum (FBS), 10 mM β-glycerol phosphate, 100 nM dexamethasone and 50 µg/ml ascorbic acid 2-phosphate, or adipogenic culture medium consisting of DMEM supplemented with 10% FBS, 1 μM dexamethasone, 10 μg/ml insulin, 0.5 mM IBMX, 60 μM indomethacin, 2 μM rosiglitazone and 20 nM IGF-1 (R&D Systems) (all Sigma unless specified). Media were replaced every 3 or 4 days. Seven days of differentiation was sufficient to assess gene expression changes in markers of differentiation. Cells were cultured for 21 days in osteoblastogenic medium to achieve fully mineralised cultures, and for 14 days in adipogenic medium for lipid production.

### Histology and biochemical analysis

Transwell discs were stained as described (Barter et al., 2015). Chondrogenic pellets and transwell discs were digested with papain (10 U/ml) at 60°C (Murdoch et al., 2007). The sulphated glycosaminoglycan (GAG) content was measured by 1,9-dimethylmethylene blue (DMB) binding (Sigma) using chondroitin sulphate (Sigma) as standard (Farndale et al., 1982), and the DNA content was measured with PicoGreen (Invitrogen) intercalating dye following the manufacturer's instructions. Cells undergoing osteoblast differentiation were fixed in 70% cold ethanol (5 min, −20°C). After drying the wells to reveal calcium-rich mineralisation deposits, the cells were incubated at room temperature with a solution of Alizarin Red (Sigma) (40 mM, pH 4.2) for 20-30 min. For quantification the staining was extracted with 10% (w/v) cetylpyridinium (Sigma) solubilised in 10 mM sodium phosphate buffer (pH 7) and the absorbance measured at 620 nM. Cells undergoing adipogenesis were fixed with formalin for 1 h, washed with distilled water and 60% isopropanol then dried. To reveal the presence of lipid droplets, the cells were stained with a 21% (w/v) solution of Oil Red O for 10 min. For quantification the staining was extracted with 100% isopropanol and the absorbance measured at 500 nM. Stained cells were washed with distilled water prior to image acquisition.

### RNA extraction and real-time reverse transcription PCR

Cartilage, synovium and fat pad samples were ground into powder and homogenised using Invitrogen TRIzol Reagent (Life Technologies) prior to RNA purification using the Qiagen RNeasy mini kit (Qiagen) essentially as previously described (Xu et al., 2012). MSC chondrogenic transwell discs were disrupted in TRIzol (for real-time RT-PCR) using a small disposable plastic pestle and an aliquot of Molecular Grinding Resin (G-Biosciences/Genotech). MSC chondrogenesis pellets were disrupted in Ambion Cells-to-cDNA II Cell Lysis buffer (Life Technologies). Total RNA was then extracted and converted to cDNA using MMLV reverse transcriptase (Invitrogen) and TaqMan real-time RT-PCR was performed and gene expression levels were calculated as described previously (Barter et al., 2010). Nuclear and cytoplasmic RNA fractions were separated using the CelLytic NuCLEAR Extraction Kit (Sigma) supplemented with RNaseOUT ribonuclease inhibitor (Life Technologies). All values are presented as the mean±s.e.m. of replicates in pooled experiments. lncRNA real-time RT-PCR amplification products were sequence verified by cloning into the pCR4-TOPO vector (Life Technologies). The Ambion FirstChoice Human Total RNA Survey Panel (AM6000) contains pools of total RNA from 20 different normal human tissues, each pool consisting of RNA from at least three tissue donors. Primer sequences are listed in Table S1.

### RNA-Seq and analysis

RNA integrity was checked using an Agilent Bioanalyzer 2100 (Agilent Technologies); RNA samples with an RNA Integrity Number (RIN)≥7 were selected. For each sample, cDNA libraries were prepared for sequencing from 5 µg of total RNA using Illumina TrueSeq mRNA kits with the manufacturers' protocols. mRNA-enriched RNA was initially purified using polydT oligo-attached magnetic beads using two rounds of purification. During the second elution the RNA was fragmented and random primed for cDNA synthesis. After the addition of a single ‘A’, base adaptors were annealed, and the products purified and enriched with PCR to create a final cDNA library. No indexing (barcoding) was performed. Library DNA size was checked using the Agilent Bioanalyzer and quantified using the Kapa Library Quant kits (Kapa Biosciences). A 7.5 pM solution of each library was loaded onto each lane of an Illumina Genome Analyzer IIa and 78-base paired-end sequencing performed. On average, each sample gave 28 million read pairs. Sample quality control was performed using FastQC (Babraham Bioinformatics). Reads were aligned to the reference genome using TopHat, specifying mate inner distance (mean inner distance between mate pairs) and standard deviation for each sample (Trapnell et al., 2012). Mapped reads were then assembled into complete transcripts using the splice junction mapping tool Cufflinks, with option –G, which utilises the Ensembl reference gene track to improve mapping. Cuffmerge was used to merge the assembled transcripts into a consensus gene track from the all of the mapped samples. Ensembl transcript biotypes were applied to identify lncRNAs (biotype lincRNA). The coding potential of lncRNAs was assessed with ORFfinder (NCBI), Coding Potential Assessment Tool (CPAT), Coding Potential Calculator (CPC) and PhyloCSF (Kong et al., 2007; Lin et al., 2011; Wang et al., 2013). RNA sequencing data have been uploaded to Gene Expression Omnibus (GEO).

### RNA-mediated interference, GapmeR transfection and lentiviral transduction

For siRNA transfection, 50 nM siRNA was transfected into 40-50% confluent MSCs using Dharmafect 1 lipid reagent (Thermo Fisher). 50 nM siRNA Dharmacon siGENOME and ON-TARGET+ siRNA (Thermo Fisher Scientific) were used to target *SOX9-AS1* and *ROCR*. Depletion of gene-specific mRNA levels was calculated by comparison of expression levels with cells transfected with 50 nM siCONTROL (non-targeting siRNA 2; 001210-02, Dharmacon). For GapmeR transfection, 100 nM Antisense LNA GapmeR (Exiqon) targeting *ROCR* or non-targeting control (Negative Control A; 300610) were transfected as for siRNAs. siRNA and GapmeR sequences are listed in Table S1. pCDH-EF1-MCS-IRES-copGFP lentivirus expression vector (System Biosciences) containing *SOX9* was generated by cloning *SOX9* from pUT-FLAG-SOX9 (Lefebvre et al., 1997). pCDH-EF1-MCS lentivirus expression vectors containing *ROCR* transcript variant 2 and transcript variant 3 were generated by cloning gBlock gene fragments (IDT) containing the sequences specified in Fig. S1. Lentiviruses expressing *SOX9*, *ROCR* or control empty vector lentivirus were generated by transfecting HEK-293T cells with pCDH plasmids, together with packaging plasmids pCMV-VSV-G (Addgene plasmid #8454, deposited by Bob Weinberg) and psPAX2 (Addgene plasmid #12260, deposited by Didier Trono). The virus-containing culture media were collected every 24 h for 3 days and concentrated (10×) with Clontech Lenti-X Concentrator into PBS (Takara). MSCs and HAC were transduced with the lentivirus-containing PBS plus 8 μg/ml polybrene. A Promega CytoTox 96 cytotoxicity assay was used to assess cell viability following siRNA treatment (Fig. S9).

### Rapid amplification of cDNA ends (RACE)

5′RACE was performed on RNA extracted from human articular cartilage or MSCs using the Invitrogen 5′ RACE System for Rapid Amplification of cDNA Ends (Life Technologies). Primer sequences are listed in Table S1. PCR amplification products were electrophoresed on agarose gels, cloned into the pCR4-TOPO vector and Sanger sequenced. The sequences have been uploaded to GenBank.

### Immunoblotting

Lysates from MSCs were prepared as described previously (Barter et al., 2010). Lysates were immunoblotted with the following antibodies: SOX9 (AB5535, 1:2000) and GAPDH (AB2302, 1:40,000) (both Merck Millipore). Secondary anti-rabbit antibodies were from Dako and chemiluminescent images were captured using a G:BOX Chemi system (Syngene).

### Statistical analysis

Data from each donor were individually analysed for gene expression and the values from each donor were then pooled to generate the mean±s.e.m. Significant differences between sample groups were assessed by one-way analysis of variance followed by the Bonferroni post-hoc test for multiple comparisons or by two-tailed Student's *t*-test for single comparisons.

## Supplementary Material

Supplementary information

Supplementary information
