# Supplementary material for: The long non-coding RNA ROCR contributes to SOX9 expression and chondrogenic differentiation of human mesenchymal stem cells
Source: Development. 2017 Dec 15;144(24):4510–21. doi: 10.1242/dev.152504 (PMC5769619; doi:10.1242/dev.152504)
Supplement: Supplementary information [file develop-144-152504-s1.pdf]

# Supplementary Figure 1

## A.

**>*ROCR* transcript variant 1 (*LOC102723505, ENST00000430908, AC005152.3*)**
AGCCTTTTAAGACACATGTTTTGGAATACAGTCAAATGCGTGCTGCTGTATGACCAGCCTATTTGAACTA
CTTAAGCATCAACTAATTAATCTGCAAGTGAAGAATACTTCCAGCATTACTCTATAATTAGACAATCTCAG
GAGGAAACAAGAGGATTTCAAAGGGGAGTCAGATGGTTTCGCAGACCGCTACATCATTTATTTTTTCATC
ACTCAGATTATGCCTCCAAATCCTACAGAAGTATAACAACCATAGAACAGACAAACACCAACAGCATCAA
CAACAGAGAAACTCAGTCCAGGAGGATATGAAACCCTTTGTAATAAAGAAAGCAACATCCATGAA

**>*ROCR* transcript variant 2**
AGTCCTTGAGGGACAAGAAAGTGGAGCCCAGGACTTTCCAGTCCCCACCTAGTGATCAACGTCTCACCC
ACGGTCAGGGGAGCAACTGGTGGTTCTACTTTTTTGAATTCTGAAGGCAAAGGCAAGCTTTTTTTAGGG
GTCCTGCTGTTGGCAGAGAAAGCTCCCAAGAGTTCTGCAGAGAAGAAGGATATAGACTCCAAAATTTTA
CTTCCAGGGACCATTTCAGTAGAGCCTTTTAAGACACATGTTTTGGAATACAGTCAAATGCGTGCTGCTG
TATGACCAGCCTATTTGAACTACTTAAGCATCAACTAATTAATCTGCAAGTGAAGAATACTTCCAGCATTA
CTCTATAATTAGACAATCTCAGGAGGAAACAAGAGGATTTCAAAGGGGAGTCAGATGGTTTCGCAGACC
GCTACATCATTTATTTTTTCATCACTCAGATTATGCCTCCAAATCCTACAGAAGTATAACAACCATAGAAC
AGACAAACACCAACAGCATCAACAACAGAGAAACTCAGTCCAGGAGGATATGAAACCCTTTGTAATAAA
GAAAGCAACATCCATGA

**>*ROCR* transcript variant 3**
CAGCGGTTGATTGCGAGGAGCAGCGGAAGGAAGGATGTATCAAACCGAAAGAAGAAAGAACCCCTGC
CAGTCGCCCCCATGACATCCGACTCTGAACGGCAGCGATCTAGAGGCTGCGGCGTGTTGCAGATTAACA
GCAGCGAGGGCAGGAGGCAAATCAGATGCCGCAGGAGCTGACTGTTTTCTTTAGCCCTGAGAGCTGAC
TGTAGGCACAGGTTGCATATCAAAGGCAACGGGAATCAGTCTGTGGCAATATTACATCAGGGACCATTT
CAGTAGAGCCTTTTAAGACACATGTTTTGGAATACAGTCAAATGCGTGCTGCTGTATGACCAGCCTATTT
GAACTACTTAAGCATCAACTAATTAATCTGCAAGTGAAGAATACTTCCAGCATTACTCTATAATTAGACAA
TCTCAGGAGGAAACAAGAGGATTTCAAAGGGGAGTCAGATGGTTTCGCAGACCGCTACATCATTTATTT
TTTCATCACTCAGATTATGCCTCCAAATCCTACAGAAGTATAACAACCATAGAACAGACAAACACCAACA
GCATCAACAACAGAGAAACTCAGTCCAGGAGGATATGAAACCCTTTGTAATAAAGAAAGCAACATCCAT
GA

## B.

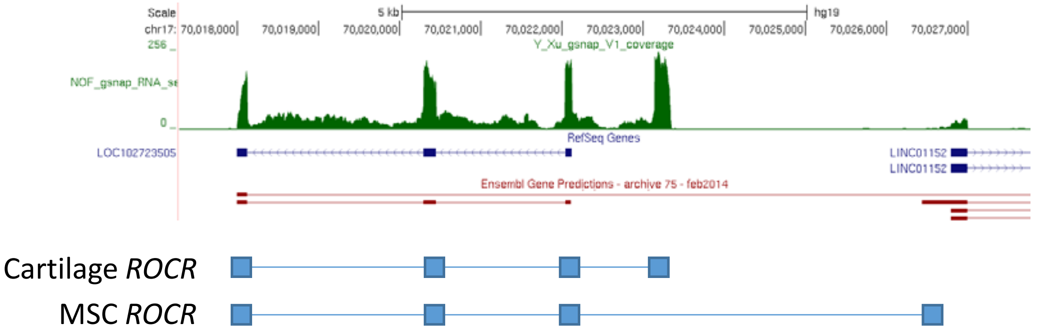


Development • Supplementary information

Supplementary Figure 1.

A. Transcript sequences of *ROCR* variants identified by RNA-Seq and RACE. Exons are delineated by the change between black and grey nucleotides. Primer locations are indicated by underlining (blue for exon1-2 assay, red for exon2-3 assay). B. Genome browser schematic of the two 4 exon transcript variants of *ROCR* with alternative first exons as indicated by 5’RACE from cartilage and MSC RNA.

# Supplementary Figure 2

## A.

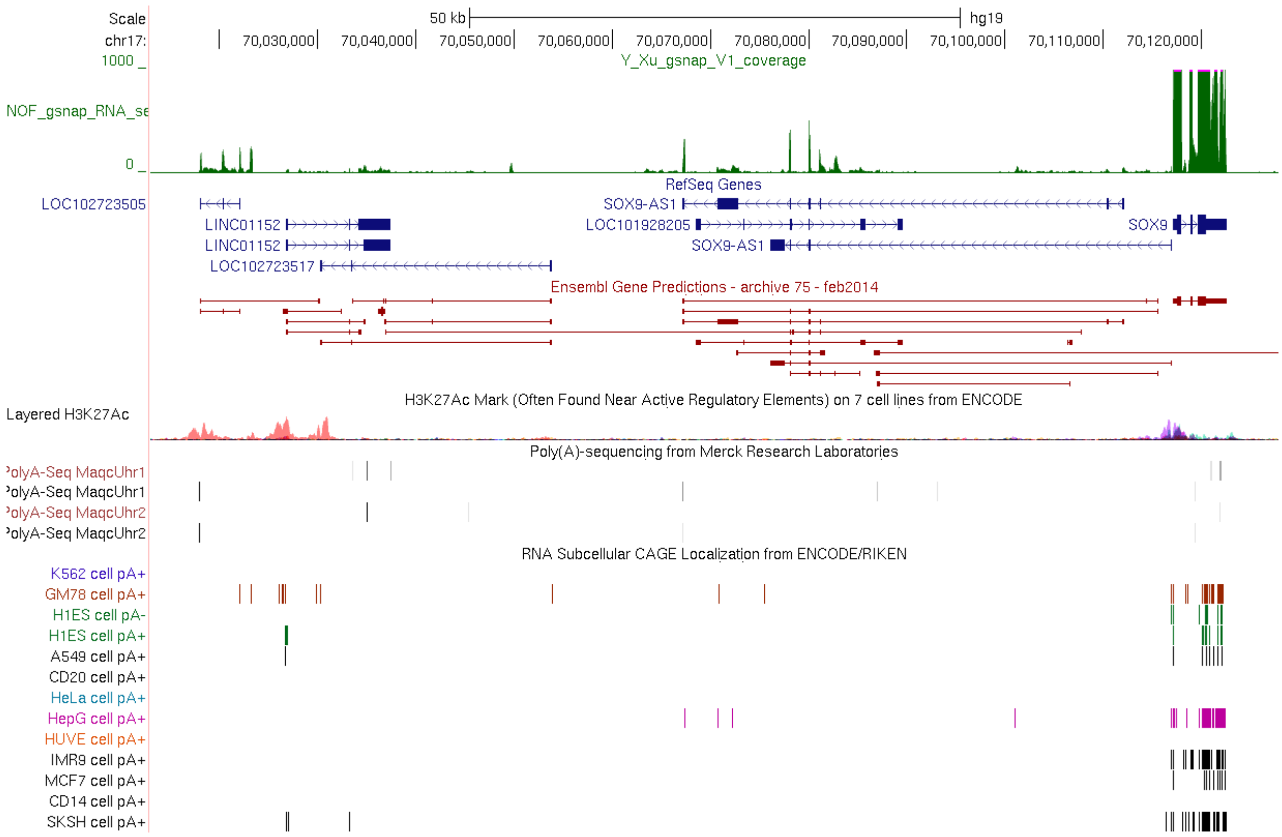


## B.

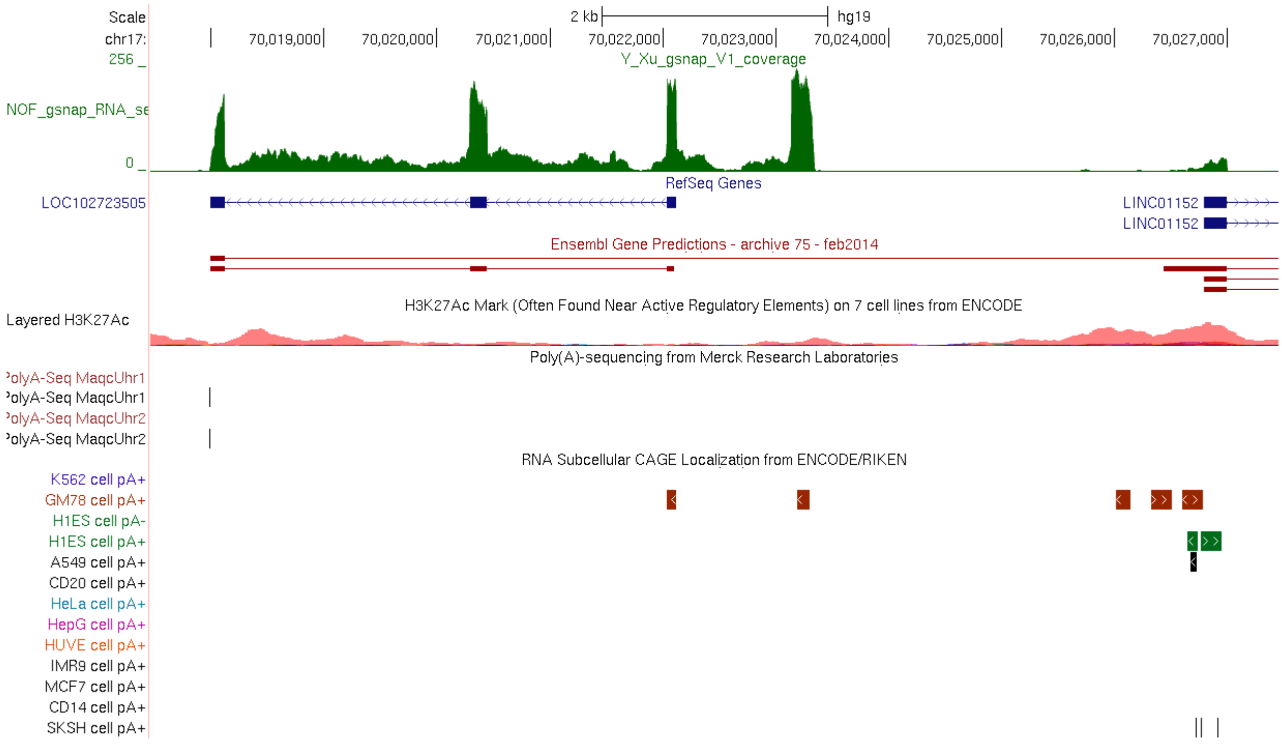


Development • Supplementary information

Supplementary Figure 2.

UCSC genome browser schematic of cartilage RNA-Seq reads aligned to the human genome with evidence of transcript start and end sites using CAGE (cap analysis gene expression) and PolyA-Seq data. A. *SOX9* and upstream locus. B. *ROCR* locus.

Development • Supplementary information

# Supplementary Figure 3

## A.

| | ORFfinder | | |
|---|---|---|---|
| | **Start** | **Stop** | **Length (nt \| aa)** |
| ENST0000430908 | 50 | 127 | 78 \| 25 |
| ROCR_HAC | 279 | 356 | 78 \| 25 |
| ROCR_MSC | 79 | 177 | 99 \| 32 |
| | 329 | 406 | 78 \| 25 |
| SOX9-AS1 | 483 | 776 | 294 \| 97 |
| | 2739 | 2930 | 192 \| 63 |
| | 837 | 1001 | 165 \| 54 |
| | 1511 | 1672 | 162 \| 53 |
| | 125 | 280 | 156 \| 51 |
| | 1393 | 1521 | 129 \| 42 |
| | 76 | 201 | 126 \| 41 |
| | 2295 | 2408 | 114 \| 37 |
| | 2100 | 2204 | 105 \| 34 |
| | 2137 | 2238 | 102 \| 33 |
| | 256 | 351 | 96 \| 31 |
| | 1908 | 1988 | 81 \| 26 |
| | 589 | 666 | 78 \| 25 |
| LINC01152 | 2605 | 2757 | 153 \| 50 |
| | 2571 | 2711 | 141 \| 46 |
| | 1048 | 1179 | 132 \| 43 |
| | 1182 | 1304 | 123 \| 40 |
| | 1967 | 2086 | 120 \| 39 |
| | 320 | 430 | 111 \| 36 |
| | 286 | 390 | 105 \| 34 |
| | 2460 | 2546 | 87 \| 28 |
| | 861 | 944 | 84 \| 27 |
| | 2251 | 2334 | 84 \| 27 |
| | 1124 | 1201 | 78 \| 25 |
| | 2803 | 2880 | 78 \| 25 |
| SOX9 | 373 | 1902 | 1530 \| 509 |
| | 1241 | 1519 | 279 \| 92 |
| | 3334 | 3564 | 231 \| 76 |
| | 2668 | 2862 | 195 \| 64 |
| | 2161 | 2322 | 162 \| 53 |
| | 2859 | 2996 | 138 \| 45 |
| | 2099 | 2227 | 129 \| 42 |
| | 1927 | 2049 | 123 \| 40 |
| | 2936 | 3037 | 102 \| 33 |
| | 3256 | 3333 | 78 \| 25 |

## B.

| | CPAT | | | | | CPC | |
|---|---|---|---|---|---|---|---|
| **Sequence Name** | **RNA Size** | **Fickett Score** | **Hexamer Score** | **Coding Probability** | **Coding Label** | **CODING POTENTIAL SCORE** | **C/NC** |
| ENST00000430908 | 346 | 0.7649 | -0.081 | 0.012 | no | -1.285 | noncoding |
| ROCR_HAC | 574 | 0.7649 | -0.081 | 0.012 | no | -0.758 | noncoding (weak) |
| ROCR_MSC | 624 | 0.7649 | -0.081 | 0.012 | no | -1.332 | noncoding |
| SOX9-AS1 | 3032 | 1.0343 | -0.010 | 0.128 | no | -1.018 | noncoding |
| LINC01152 | 3578 | 0.9027 | -0.053 | 0.015 | no | 0.364 | coding (weak) |
| SOX9 | 3963 | 1.3018 | 0.647 | 1.000 | yes | 8.199 | coding |

Development • Supplementary information

# Supplementary Figure 3

C.

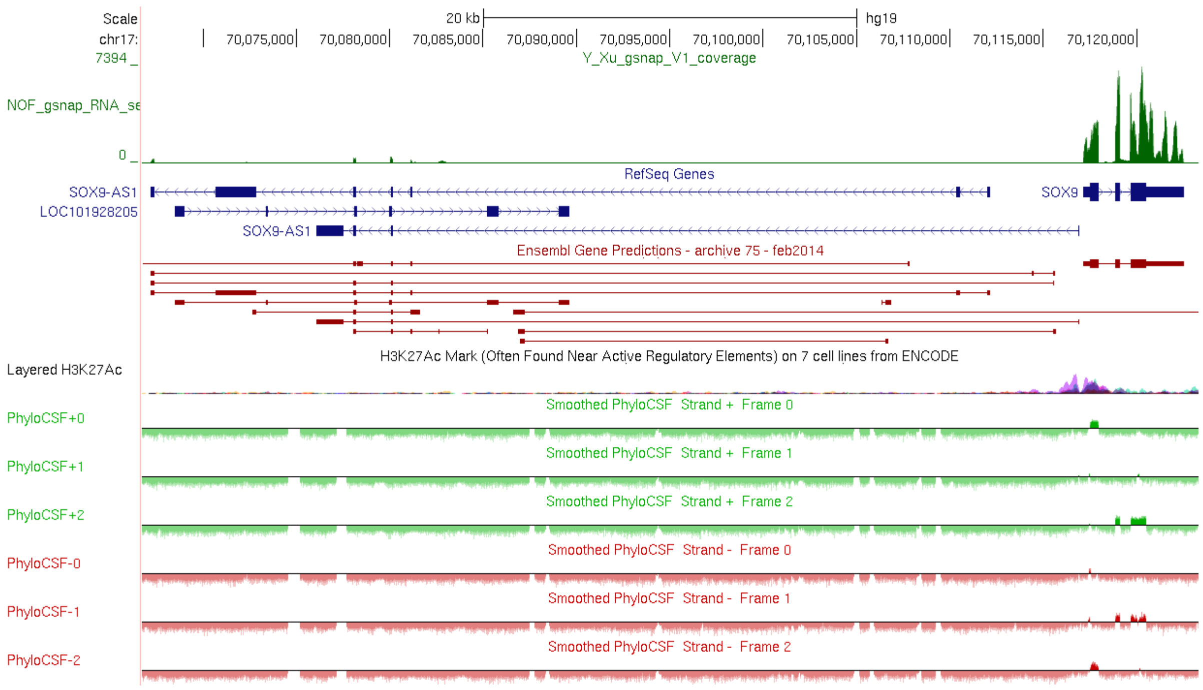


D.

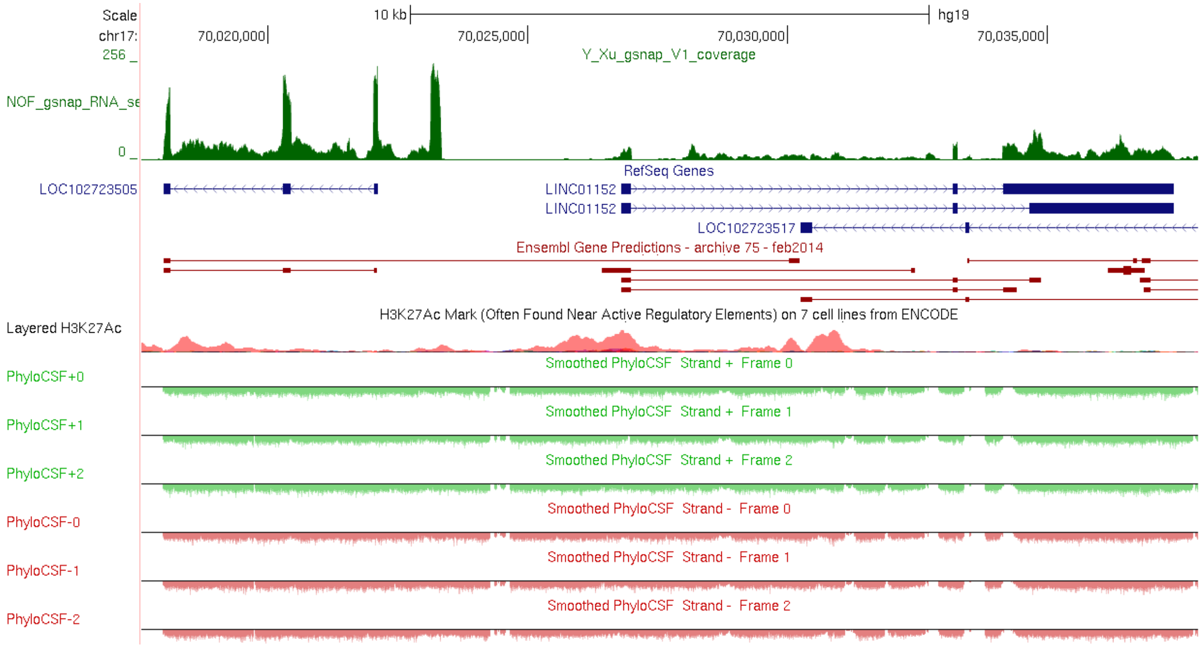


Development • Supplementary information

Supplementary Figure 3.

*In silico* analysis indicates a lack of coding potential for both *SOX9-AS1* and *ROCR*, with the existence of A. only very short open reading frames (ORF Finder) and B-D. codon substitution rates indicative of noncoding transcripts. B. CPAT and CPC. C. PhyloCSF on *SOX9* and *SOX9-AS1* locus. D. PhyloCSF on *ROCR* locus.

# Supplementary Figure 4

A.

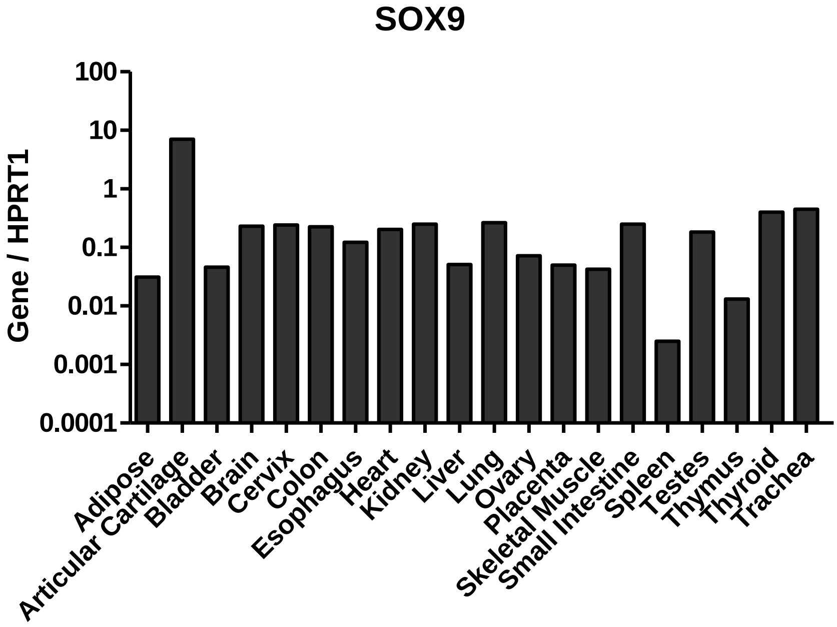


B.

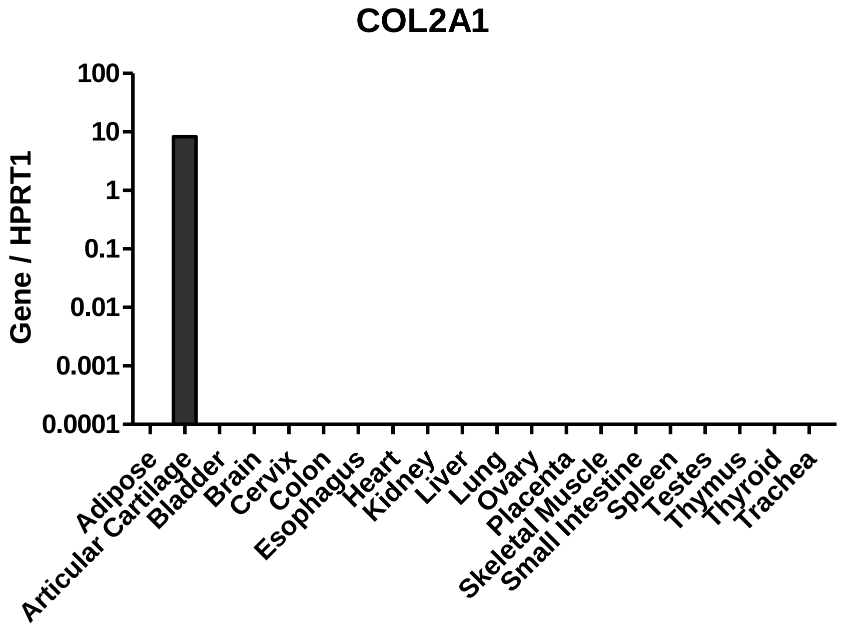


C.

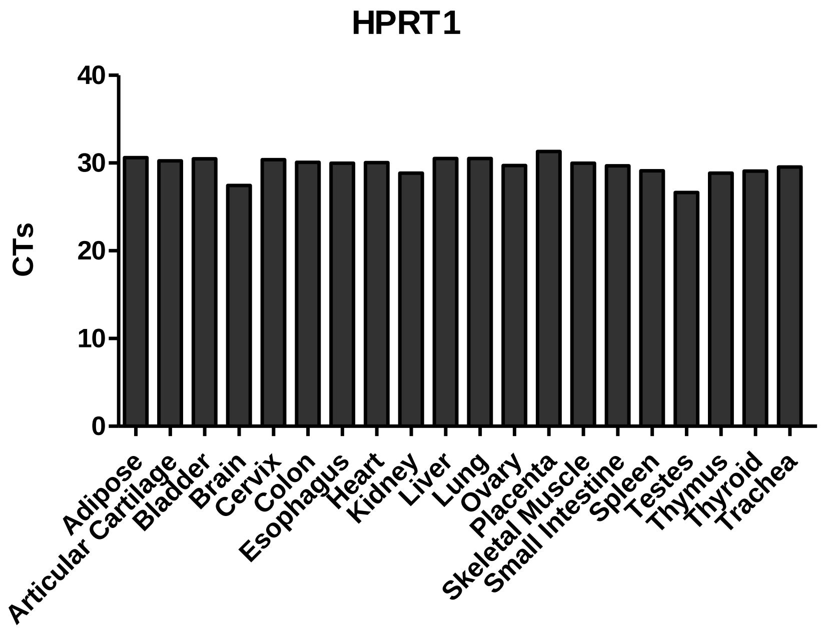

Supplementary Figure 4.

Expression of A. *SOX9*, B. *COL2A1* and C. *HPRT1* in an RNA tissue panel measured by real-time RT-PCR. Values are the technical mean of data pooled from 3 donors per tissue.

Development • Supplementary information

# Supplementary Figure 5

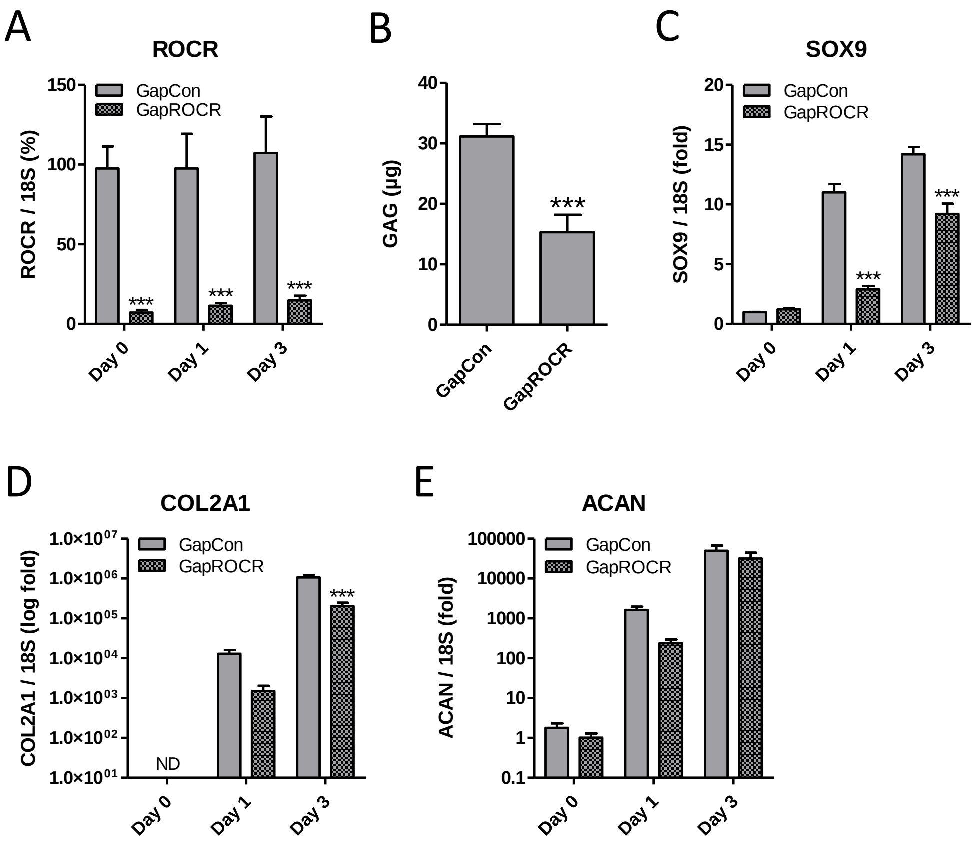


Supplementary Figure 5.

MSCs were transfected for 1 day with *ROCR*-targetting or non-targetting control GapmeR prior to chondrogenic differentiation in a V-bottom 96 well plate. A. *ROCR* expression in RNA extracted from Day 0, 1 and 3 chondrogenic pellets. Expression is presented as a percentage of non-targetting control levels. B. GAG levels assayed by DMB assay in Day 7 chondrogenic pellets. C-E. Expression of (C) *SOX9*, (D) *COL2A1*, (E) *ACAN* in RNA extracted from Day 0, 1 and 3 chondrogenic pellets. Values are the mean ± SEM of data pooled from 4 MSC donors. *** = $P < 0.001$ for *ROCR* GapmeR versus non-targetting GapmeR.

# Supplementary Figure 6

A

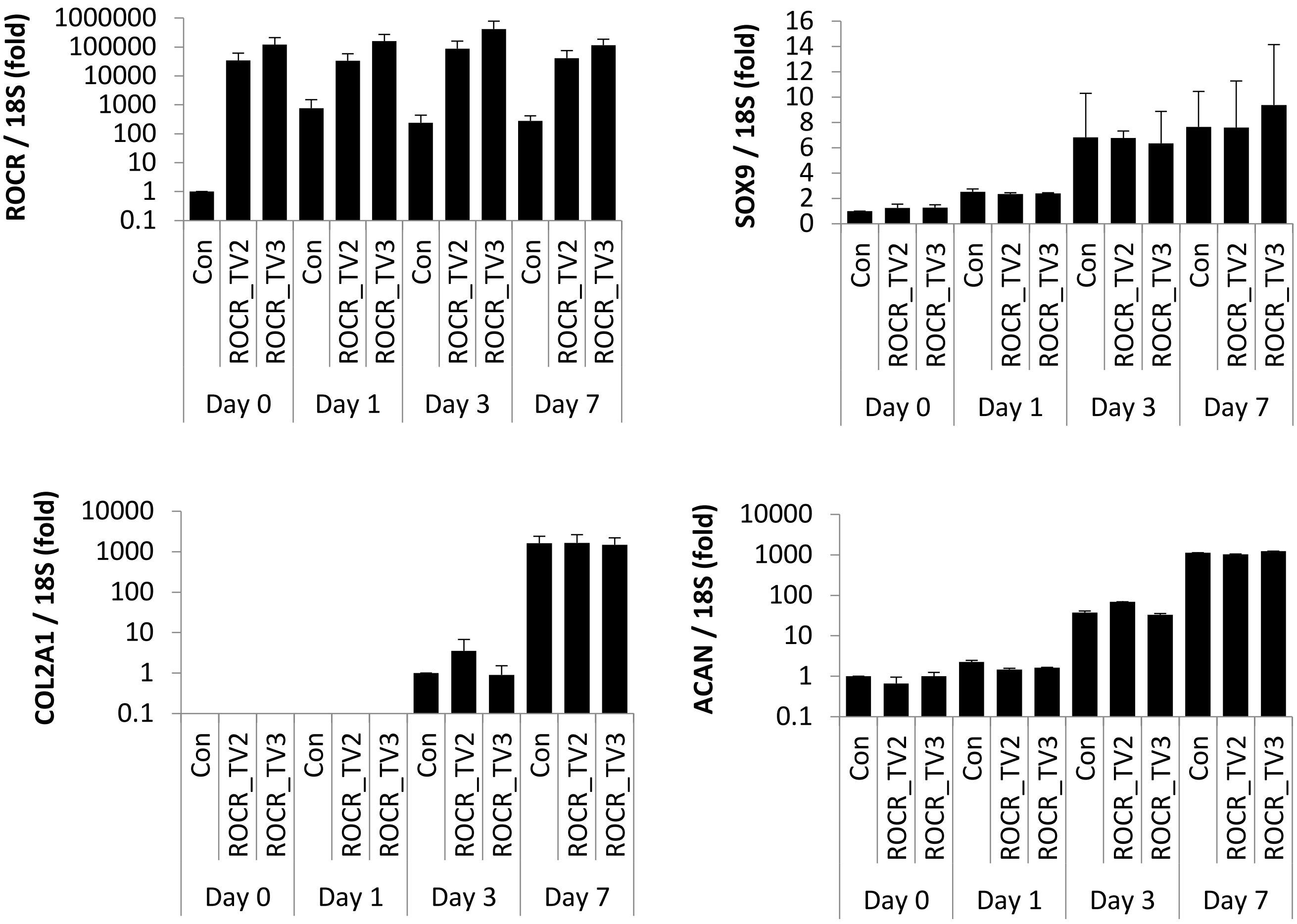


B

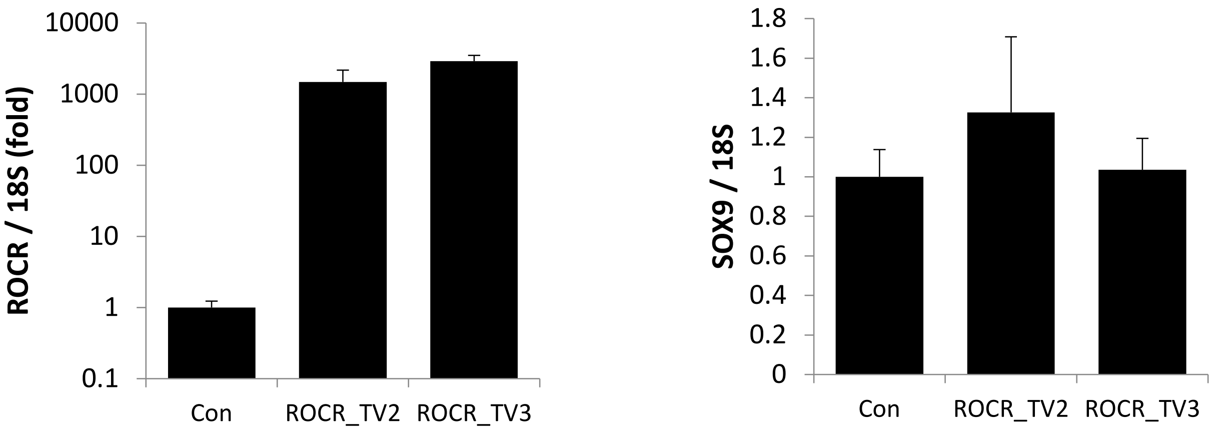


Development • Supplementary information

Supplementary Figure 6.

A. MSC were transduced for 2 days with *ROCR*-expressing lentivirus particles (transcript variant 2 and 3) prior to chondrogenic differentiation for up to 7 days. Expression of *ROCR*, *SOX9*, *COL2A1* and *ACAN* in RNA extracted from Day 0, 1, 3 and 7 chondrogenic pellets. Values are the mean ± SEM of data pooled from 2 MSC donors. B. *ROCR* and *SOX9* expression in HAC transduced with *ROCR*-expressing lentivirus particles (transcript variant 2 and 3) for 3 days. Values are the mean ± SEM of data pooled from 2 OA HAC donors.

Development • Supplementary information

# Supplementary Figure 7

A.

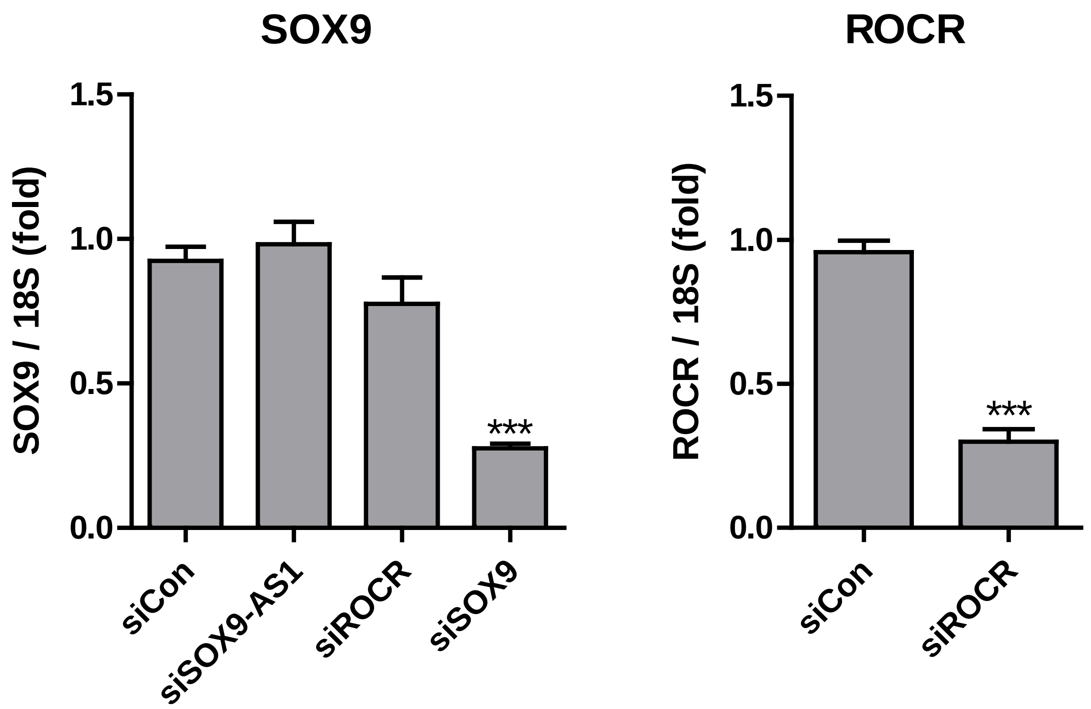


B.

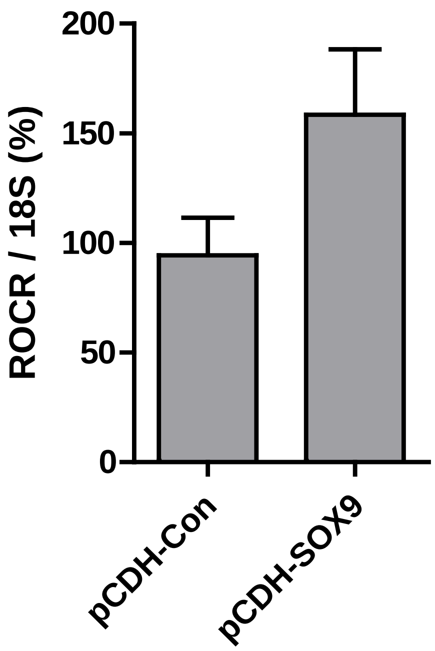


Supplementary Figure 7.

A. *SOX9* and *ROCR* expression in HAC transfected for 2 days with the indicated siRNAs. Values are the mean ± SEM of data pooled from 4 OA HAC donors. *** = P < 0.001. B. *ROCR* expression in MSCs transduced with SOX9 or control lentivirus (pCDH) for 2 days. Values are the mean ± SEM of data pooled from 4 MSC donors.

# Supplementary Figure 8

A.

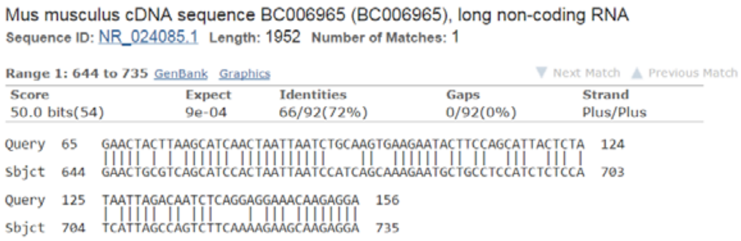


C.

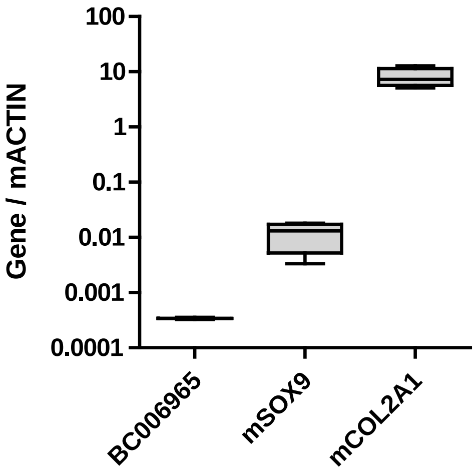


B.

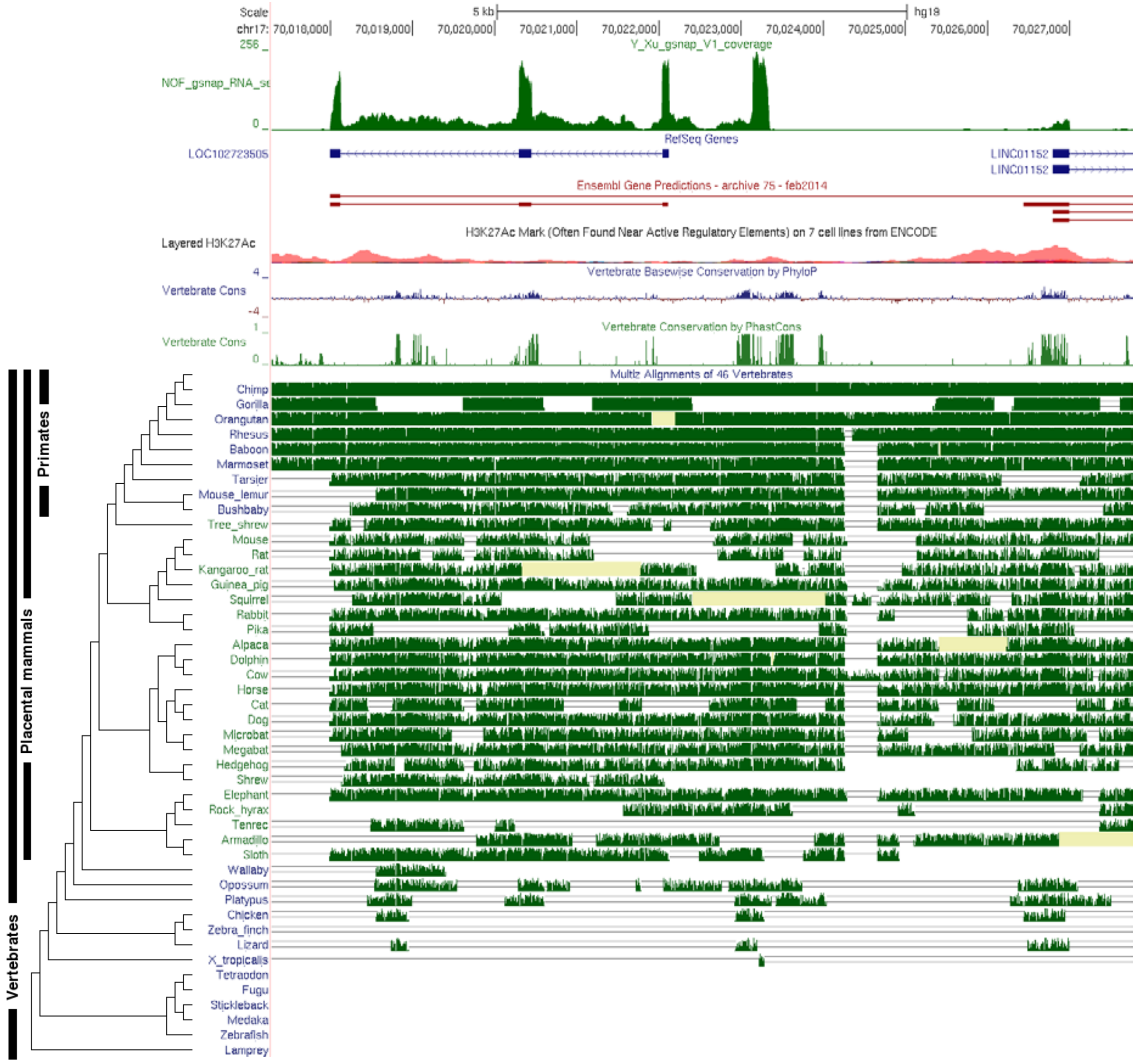


Development • Supplementary information

Supplementary Figure 8.

A. Blastn result of homology search for *ROCR* sequence in mouse. B. UCSC genome browser schematic of the *ROCR* locus showing vertebrate conservation. C. Expression of *Bc006965*, *Sox9* and *Col2a1* in C57BL/6 mouse xiphoid cartilage RNA.

Development • Supplementary information

# Supplementary Figure 9

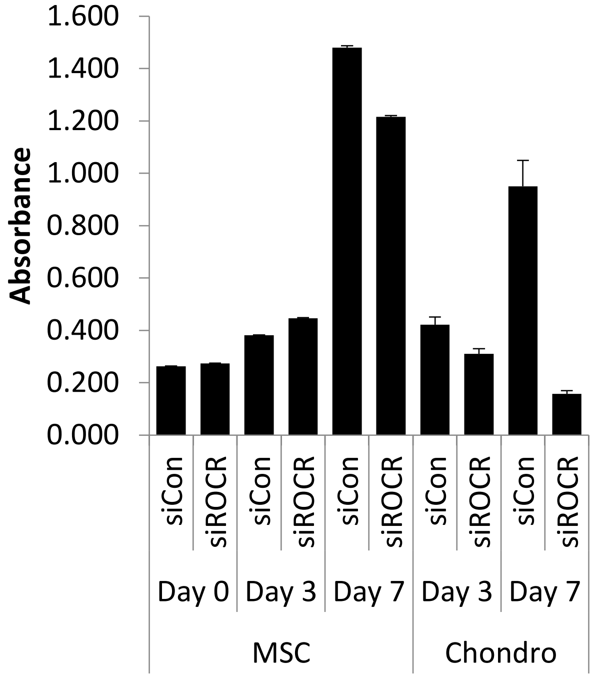


Supplementary Figure 9.

MSCs were transfected for 2 days with *ROCR*-targetting or non-targetting control siRNA prior to chondrogenic differentiation in a V-bottom 96 well plate or maintenance in MSC medium for up to 7 days. Media were collected immediately prior to the induction of chondrogenesis (Day 0) and after 3 and 7 days. Cytotoxicity was measured with the use of the Promega CytoTox 96 kit.

Supplementary Table 1.

Primer and siRNA/GapmeR sequences.

| Primer | Sequence | Probe |
|---|---|---|
| SOX9-AS1 exon4-5 F | ccatgagcatttctcgtctg | 65 |
| SOX9-AS1 exon4-5 R | caggacaaggtggatgaaca | |
| SOX9-AS1 exon5-6 F | ctggcaagaggcaggaaa | 38 |
| SOX9-AS1 exon5-6 R | gctctcatccaaggtgaacag | |
| ROCR exon2-3 F | tgctgtatgaccagcctatttg | 11 |
| ROCR exon2-3 R | tcctcttgtttcctcctgaga | |
| ROCR exon1-2 F | ctgaaggcaaaggcaagc | 18 |
| ROCR exon1-2 R | caaaacatgtgtcttaaaaggctct | |
| LINC01152 F | cctcacttcctcctcttcctg | 19 |
| LINC01152 R | ccccatctcatctgtttttga | |
| RUNX2 F | gtgcctaggcgcatttca | 29 |
| RUNX2 R | gctcttcttactgagagtggaagg | |
| ALPL F | agaaccccaaaggcttcttc | 31 |
| ALPL R | cttggcttttccttcatggt | |
| ADIPOQ F | ggtgagaagggtgagaaagga | 85 |
| ADIPOQ R | tttcaccgatgtctcccttag | |
| FABP4 F | ccaccataaagagaaaacgagag | 31 |
| FABP4 R | gtggaagtgacgcctttcat | |
| L-SOX5a F | tttacctcaggagtttgaaagga | 38 |
| L-SOX5a R | gcttgtcaccatggctacct | |
| SOX6 F | gcttctggactcagccctttta | 67 |
| SOX6 R | ggccctttagcctttggtta | |
| | | |
| ROCR RACE GSP1 | TTTGAAATCCTCTTGTTTC | |
| ROCR RACE GSP2 | CAAAACATGTGTCTTAAAAGGCTCT | |
| | | |
| siRNA | Target sequence | |
| siSOX9-AS1 | CAAAGGAGCTAAAGAAGAA | |
| siROCR-1 | GGAGGAAACAAGAGGATTT | |
| siROCR-2 | CAACCATAGAACAGACAAA | |
| | | |
| GapmeR | Target sequence | |
| GapROCR | ACTTAAGCATCAACTA | |

Supplementary Table 2.

Cartilage RNA-Seq lncRNA expression and *SOX9* expression.

Click here to Download Table S2

Supplementary Table 3.

Expression of *ROCR* and *SOX9-AS1* in cell types from Human Protein Atlas and Illumina BodyMap RNA-Seq data.

Click here to Download Table S3
